# Adaptation and Validation of the Gluten-Free Perceived Nutrition Environment Measures Survey (NEMS-P-GF) and Its Association with Gluten-Free Diet Adherence Among Adults with Celiac Disease in Chile

**DOI:** 10.3390/nu18060929

**Published:** 2026-03-16

**Authors:** María Jesús Vega-Salas, Alejandra Parada, Danae Hermosilla-Llanca, Loni Berkowitz, Lorena Rodríguez Osiac, Daniel Egaña Rojas, Attilio Rigotti

**Affiliations:** 1 Departamento de Nutrición y Dietética, Escuela de Ciencias de la Salud, Facultad de Medicina, Pontificia Universidad Católica de Chile, Santiago 7820436, Chile; muvega@uc.cl (M.J.V.-S.); danae.hermosilla@alumni.uc.cl (D.H.-L.); 2 Center for Cancer Prevention and Control (CECAN), Santiago 7820436, Chile; 3 Departamento de Nutrición, Diabetes y Metabolismo, Escuela de Medicina, Pontificia Universidad Católica de Chile, Santiago 7820436, Chile; lberkowi@uc.cl (L.B.); arigotti@uc.cl (A.R.); 4 Centro de Nutrición Molecular y Enfermedades Crónicas, Escuela de Medicina, Pontificia Universidad Católica de Chile, Santiago 8331010, Chile; 5 Escuela de Salud Pública Dr. Salvador Allende, Universidad de Chile, Santiago 8380453, Chile; lorenarodriguez@uchile.cl; 6 Departamento de Atención Primaria y Salud Familiar, Facultad de Medicina, Universidad de Chile, Santiago 8900085, Chile; degana@uchile.cl

**Keywords:** celiac disease, gluten-free diet, food environments, dietary adherence, structural determinants of diet

## Abstract

**Background/Objectives:** Strict adherence to a gluten-free diet (GFD) is the only effective treatment for celiac disease (CeD) but remains challenging due to structural and environmental barriers. Evidence on these determinants in Latin America is scarce. This study aimed to adapt and validate the Gluten-Free Perceived Nutrition Environment Measures Survey (NEMS-P-GF) for adults with CeD in Chile and examine its association with GFD adherence. **Methods:** A cross-sectional online survey (October 2023–January 2024) included adults (≥18 years) with biopsy- or serology-confirmed CeD (n = 233). The questionnaire collected sociodemographic and clinical data, assessed adherence using the Celiac Dietary Adherence Test (CDAT; good < 13, poor ≥ 13), and measured perceptions of home and supply food environments via the adapted NEMS-P-GF. Construct validity was tested using exploratory factor analysis and reliability with Cronbach’s α and McDonald’s ω. Associations with adherence were analyzed using Mann–Whitney U. **Results:** NEMS-P-GF domains showed adequate validity (KMO 0.71–0.81; Bartlett’s *p* < 0.001) and acceptable-to-excellent reliability (α/ω = 0.70–0.90). Participants with good vs. poor adherence perceived more supportive environments, particularly at home (median 4.79 vs. 1.29; *p* < 0.01) and globally (1.72 vs. −7.25; *p* < 0.01). Supply environments were perceived as less supportive due to limited availability and high prices (median −3.68 and −7.78), with smaller differences between adherence groups (*p* = 0.018). **Conclusions:** Supportive home environments were strongly associated with better GFD adherence, while supply environments remained broadly restrictive, showing modest but significant differences between adherence groups. The NEMS-P-GF demonstrated preliminary evidence of good psychometric properties and offers a valid, context-sensitive tool to assess GF food environments and inform public health strategies for CeD populations.

## 1. Introduction

Celiac disease (CeD) is a chronic autoimmune gastrointestinal disorder triggered by gluten ingestion in genetically susceptible individuals. Pooled evidence from a systematic review and meta-analysis of studies published between 1991 and 2016 estimates that CeD affects approximately 1–2% of the global population, with prevalence increasing worldwide and varying by age, sex, and region [1,2]. Gluten exposure among people with CeD induces an immune response that causes intestinal inflammation, villous atrophy, and impaired nutrient absorption. These changes lead to a wide range of gastrointestinal and extraintestinal manifestations, such as diarrhea, abdominal discomfort, anemia, osteoporosis, and neurological complications [3]. Individuals with CeD often exhibit alterations in gut microbiota and face an increased risk of gastrointestinal malignancies, including small bowel adenocarcinoma and intestinal lymphomas [4,5,6]. These long-term complications underscore the importance of early diagnosis and sustained treatment to achieve optimal health outcomes and quality of life.

The only effective treatment for CeD is a strict, lifelong adherence to a gluten-free diet (GFD), which promotes intestinal healing and prevents complications such as nutrient deficiencies, bone disease, and malignancies [7]. Yet, adherence rates vary considerably—from 42% to 91%—depending on population characteristics and assessment methods [8]. While individual factors such as motivation, symptom relief, and disease knowledge play a role, growing evidence underscores the influence of social, economic, and environmental conditions on the ability to maintain a GFD [9,10,11]. Structural obstacles, such as the limited availability and high prices of gluten-free foods (GFF), pose significant challenges, particularly for individuals with lower socioeconomic status or food insecurity. These constraints affect not only dietary adherence but also overall well-being and social inclusion [12,13].

In Chile, CeD affects about 1% of adults, with a higher prevalence among women [14]. Earlier studies reported a bimodal age distribution at diagnosis, with peaks in early childhood and young adulthood [15]. More recent evidence shows that adults are typically diagnosed around age 40, often with complications such as anemia or elevated liver enzymes [16]. Historically associated with underweight, CeD now increasingly coexists with overweight and obesity, reflecting complex interactions between genetic factors, intestinal changes, microbiota alterations, and social factors [17,18]. In this context, epidemiology shift underscores the need to examine not only adherence to a GFD but also the nutritional quality of GFF [19], as increased reliance on processed GFF may contribute to unfavorable dietary patterns and weight gain [20,21]. Despite growing attention to the nutritional quality of GFD worldwide, research on how structural and social determinants influence adherence and nutritional outcomes in CeD remains limited.

Food environments—defined as the physical, sociocultural, economic, and political contexts that shape the availability, accessibility, affordability, and appeal of foods—play a critical role in influencing dietary behaviors [22]. Research on the global obesity epidemic demonstrates that obesogenic environments, which promote abundant, low-cost, energy-dense, nutrient-poor foods, foster unhealthy eating patterns and widen nutrition-related health inequities [23]. These same environmental conditions constrain adherence to medically prescribed diets such as the GFD. Individuals with CeD face limited access to certified GFF, higher costs relative to gluten-containing products, inadequate labeling standards, and greater exposure to cross-contamination risks [24,25]. Barriers are further exacerbated among vulnerable populations experiencing food insecurity, limiting their access to safe and adequate foods [26].

Despite advances in food environment research, its application to CeD remains limited. Supporting a nutritionally adequate GFD poses unique challenges: GF processed products tend to be higher in fat, sodium, sugar, and refined starches, and lower in fiber, minerals, and vitamins, which can further disrupt the gut microbiota and impair intestinal health [27,28,29]. Nutritionally balanced GFDs require adequate intake of naturally GFF, such as fruits, vegetables, legumes, fish, meat, and whole grains, which are themselves influenced by the environments in which people access and prepare food [30]. Understanding these environmental determinants is therefore essential for developing strategies to improve GFD adherence and overall dietary quality, contributing to equity in nutritional care.

Only a few studies have explored the role of food environments in CeD. Cyrkot et al. (2022) examined home food environments and purchasing behaviors among Canadian youth with CeD [31], adapting items from the Perceived Nutrition Environment Measures Survey (NEMS-P) [32]. However, their instrument did not distinguish between gluten-free (GF) and gluten-containing foods and lacked a composite score to evaluate the overall supportiveness of the domestic food environment for individuals with CeD. Moreover, the tool lacked validation for use in CeD populations. In Chile, the NEMS-P was culturally adapted and validated as the NEMS-P-Ch, yielding a reliable and internally consistent 48-item instrument capturing perceptions of home, street, restaurant, and supply environments [33]. Yet, neither the original NEMS-P nor the Chilean adaptation was designed to assess perceptions of GFF environments among people who require a medically prescribed GFD. To date, no studies have examined the relationship between food environments and CeD in Latin America, and evidence from Chile is virtually nonexistent, underscoring a significant gap in the literature with implications for policy and practice.

To address this gap, the present study adapted the NEMS-P-Ch instrument to develop the Gluten-Free Perceived Nutrition Environment Measures Survey (NEMS-P-GF), which captures the perceptions of the CeD population regarding the availability, accessibility, and affordability of GF and gluten-containing foods within both home and supply environments. This tool offers a context-specific approach to understanding how structural and perceptual dimensions of food environments influence adherence to a GFD.

Accordingly, the aims of this study were twofold: (1) to adapt and validate the NEMS-P-GF instrument for adults with CeD in Chile, and (2) to examine the relationship between perceived GFF environments and adherence to a GFD. By integrating the study of food environments with clinical and nutritional considerations, this work contributes to emerging international efforts to support strategies aimed at improving the food environments for CeD populations in Chile and similar contexts, ultimately minimizing further health complications to CeD populations.

## 2. Materials and Methods

### 2.1. Study Design and Participants

This study employed a cross-sectional design, utilizing an online questionnaire distributed between October 2023 and January 2024 through a public invitation disseminated via social media platforms affiliated with Fundación Convivir, a non-governmental organization that advocates for individuals with CeD. Eligible participants were adults (≥18 years) residing in Chile with a medical diagnosis of CeD confirmed by a physician and by serology and/or biopsy. Participation was voluntary, and informed consent was obtained before survey initiation. The study protocol was approved by the Health Sciences Ethics Committee at Pontificia Universidad Católica de Chile (ID# 230320004). All procedures were conducted under the ethical standards of the Helsinki Declaration. This study is reported in accordance with the STROBE checklist for cross-sectional studies [34] and the COSMIN guidelines for the selection and evaluation of measurement properties of patient-reported outcome measures [35].

### 2.2. Data Collection

The questionnaire was designed to collect data across several domains relevant to the study objectives, including:

- Sociodemographic and anthropometric characteristics: age, gender, education level, household socioeconomic status (SES) [36], self-reported weight and height.
- Clinical history of celiac disease: time since diagnosis, diagnostic method.
- Adherence to a gluten-free diet (GFD): assessed using the validated Celiac Dietary Adherence Test, a 7-item self-report questionnaire for assessing GFD adherence in clinical and research settings (CDAT; score range 7–35, higher scores indicate poorer adherence) [37,38]. Participants were classified into two adherence groups: Good (<13 points) and Poor (≥13 points), based on the classification used in previous Chilean GFD adherence studies [11].
- Perceptions of the gluten-free food (GFF) environment in the home and in stores, particularly regarding the availability, accessibility, and affordability of GF and gluten-containing products (NEMS-P-GF). Table 1 summarizes the dimensions of the adapted NEMS-P-GF.

**Figure 1:**
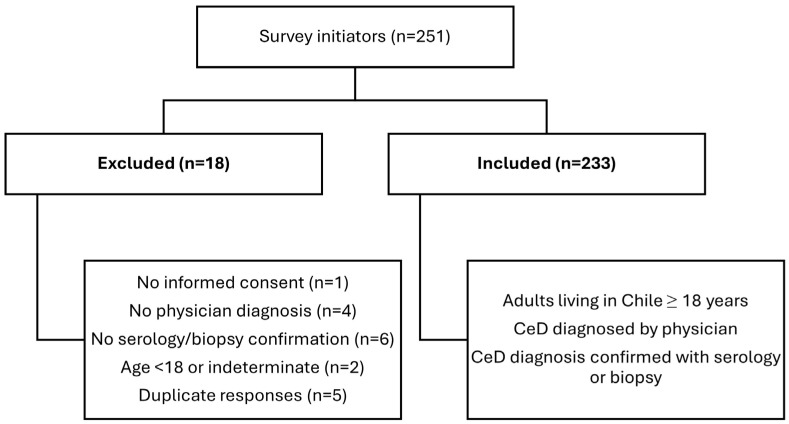
Flow diagram of participant inclusion and exclusion.

The questionnaire was administered using Google Forms. Following data cleaning and quality control procedures, a total of 233 valid responses were retained for analysis (Figure 1). Due to platform constraints, it was not possible to distinguish between survey link views and initiated responses. This sample size meets commonly accepted thresholds for psychometric evaluation using exploratory factor analysis, including assessment of structural validity and internal consistency [39], and is adequate for exploratory analyses examining moderate differences in NEMS-P-GF scores between adherence groups.

### 2.3. Adaptation, User Validation, and Pilot Testing

The NEMS-P-GF was developed through a staged adaptation of the NEMS-P-Ch [33], supplemented with items and wording approaches from the gluten-free home environment adaptation by Cyrkot et al. (2022) [31]. First, we mapped NEMS-P-Ch items to the target domains (home and food supply) and classified them as retained, modified, removed, or added to ensure relevance for adults with CeD. Modifications captured features specific to GFF environments, including the relative availability of GFF versus gluten-containing products, access constraints, and price differentials, resulting in a preliminary NEMS-P-GF questionnaire. Second, to generate content validity evidence, experts in food environments and CeD reviewed item relevance, clarity and alignment of response options, and cultural appropriateness for Chile. Third, user validation and pilot testing with ten adults with CeD assessed comprehension of key terms and comparative phrasing (GFF vs. gluten-containing) and usability of the online response scales. Feedback informed minor refinements to wording, item order, and response categories. The final instrument version used in this study is provided in the Supplementary Materials (Questionnaire).

### 2.4. Data Analysis

Analyses were conducted in three stages: (i) preliminary psychometric evaluation of the adapted NEMS-P-GF; (ii) descriptive statistics; and (iii) inferential analysis of associations with GFD adherence. Data were analyzed using Jamovi (v2.4.14; R-based backend).

#### 2.4.1. Instrument Validation

To evaluate the psychometric properties of the adapted NEMS-P-GF, we assessed structural validity and internal consistency reliability, in line with COSMIN-recommended measurement properties for instrument validation. The scoring system was adapted from the NEMS-P-Ch framework to maintain the directional consistency of values, with higher scores reflecting a more supportive GFF environment [40].

Several NEMS-P-GF components reflect different measurement model types. Perceived environment subscales (e.g., availability, accessibility, and price perceptions) were treated as reflective indicators of an underlying perceived supportiveness dimension and were therefore evaluated using exploratory factor analysis (EFA) and internal consistency. In contrast, checklist/condition-based components (e.g., kitchen appliances, food purchase attributes, and family commensality) were treated as descriptive indicators.

Structural validity was examined using exploratory factor analysis (EFA), conducted separately for each subscale due to differing response formats and domain-specific item sets. EFA used the minimum residual extraction method and Varimax rotation, selected for interpretability and comparability with the NEMS-P-Ch scoring workflow [40]. The number of factors retained within each subscale was determined using the Kaiser criterion (eigenvalues > 1) and interpretability against the instrument’s conceptual framework. Sampling adequacy was assessed using the Kaiser–Meyer–Olkin (KMO) statistic and Bartlett’s test of sphericity (KMO > 0.60; *p* < 0.05). Items with rotated loadings < 0.30 were considered for exclusion unless retained on conceptual grounds.

Item responses were recoded to a standardized −2 to +2 scale, with higher values indicating a more supportive gluten-free food (GFF) environment; reverse-worded items were inverted. Weighted subscale scores were calculated by multiplying each retained item’s recoded value by its corresponding factor-loading weight and summing across items. Items administered but showing non-salient/zero loadings were not included in the factor-derived scores and were treated as descriptive indicators, prioritized for refinement/expansion in future work. Subscale scores were then aggregated to derive domain-level (home and supply) and global NEMS-P-GF scores. By construction, 0 represents the neutral/midpoint response across items; therefore, values above 0 indicate a net perception of a more supportive environment and values below 0 indicate a net perception of a more restrictive environment.

Internal consistency was assessed using Cronbach’s alpha and McDonald’s omega (ω) for each retained NEMS-P-GF subscale, with coefficients ≥ 0.70 considered acceptable.

#### 2.4.2. Descriptive Statistics

Participant characteristics and study variables were summarized using means (SD) or medians (P25–P75) for continuous variables and frequencies (percentages) for categorical variables. Variables included sociodemographic and anthropometric data, CDAT scores, and NEMS-P-GF domain and global scores.

#### 2.4.3. Inferential Analysis

Associations between GFD adherence (Good vs. Poor, per CDAT) and explanatory variables were examined using chi-square for categorical variables and *t*-tests or non-parametric equivalents (Mann–Whitney U) for continuous variables, as appropriate. A significance level of α = 0.05 was used for all tests. To complement significance testing, effect sizes were calculated for between-group comparisons: Cohen’s *d* for *t*-tests and effect size *r* (Z/√N) for Mann–Whitney U tests, with thresholds of 0.10, 0.30, and 0.50 interpreted as small, moderate, and large effects, respectively.

## 3. Results

### 3.1. Participant Characteristics

A total of 233 adults with biopsy and/or serology-confirmed CeD completed the survey. Adherence groups were balanced (*p* = 0.471): 52.4% showed good adherence (CDAT < 13). Participants had a mean age of 38.6 years, and 90% were female. University and postgraduate education accounted for 37.5% and 28.0%, respectively; most households were high SES (68.2%). Mean BMI was 24.8 kg/m^2^. Over half (56.7%) reported ≥3 years since diagnosis, and 13.7% had another household member with CeD. No significant differences were observed between adherence groups for sociodemographic, anthropometric, or clinical variables (all *p* > 0.05) (Table 2).

### 3.2. Construct Validity and Internal Consistency Reliability of the NEMS-P-GF

Exploratory factor analyses showed adequate sampling for all targeted subscales (KMO 0.71–0.81; all Bartlett *p* < 0.001), except for “Family commensality”, “Kitchen appliances”, and “Food purchase attributes” (all KMO < 0.6). These components are checklist/condition-based indicators rather than reflective latent subscales; therefore, low factorability and weak/zero loadings are expected and are not interpreted as evidence of poor constructs. Consistent with this conceptualization, they were not retained as latent subscales for the factor-derived domain/global composite scores (Supplementary Table S1). Within the home environment, three factors were identified for food availability (naturally GFF; GF processed products; gluten-containing processed products), with moderate-to-high loadings (0.47–0.86). Supply-environment analyses replicated three-way structures for in-store accessibility and prices (loadings 0.57–0.99). Neighborhood accessibility separated into naturally GFF versus GFF-specific availability/quality/variety (loadings 0.80–0.94). Criteria for choosing stores were separated into social/spatial proximity versus selection/quality/price; one item (public transportation) showed no loading and was excluded (KMO = 0.65).

Internal consistency reliability was acceptable to excellent for most retained subscales (e.g., α or ω ≈ 0.70–0.90) (Table 3). Within the home environment, the overall availability of foods showed acceptable consistency (α = 0.62–0.88). In the supply environment, neighborhood accessibility and in-store GFF accessibility demonstrated good reliability (α = 0.79–0.90), whereas food price subscales ranged from acceptable to excellent (α = 0.77–0.91). Both kitchen appliances (α = 0.35) and purchase attributes (α = 0.55) showed poor internal reliability, consistent with the findings from the EFA. Therefore, they were excluded from the later composite scores by food environments and global NEMS-P-GF scores.

### 3.3. NEMS-P-GF Domains and Global Scores

Most participants reported negative global NEMS-P-GF scores (weighted scores) (median −2.88), indicating that, overall, GFF environments were perceived as less supportive. Conversely, the home environment was perceived as relatively supportive (median 2.83), whereas the supply environment remained unsupportive (median −6.65) (Supplementary Table S2). Within the home environment domain, positive perceptions were driven by the availability of naturally GFF and GF-processed products (median 1.96 and 1.59, respectively). Supply-environment limitations stemmed primarily from low neighborhood accessibility to GF-specific products (median −1.90) and low availability and high prices for GFF-processed items (median −0.58 and −6.55, respectively).

### 3.4. NEMS-P-GF Scores by GFD Adherence

**Figure 2:**
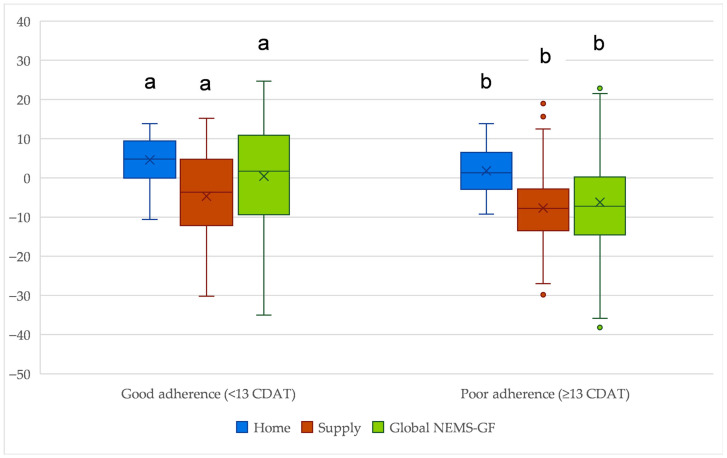
Weighted NEMS-P-GF scores (Home, Supply, Global) according to adherence to a gluten-free diet (CDAT). Boxplots show medians, interquartile ranges, whiskers (1.5 × IQR), and means (X). Statistical differences between adherence groups (Good < 13 vs. Poor ≥ 13) were assessed using the Mann–Whitney U test: Home < 0.01; Supply = 0.018; Global < 0.01; Superscript letters indicate statistically significant differences between adherence groups within each domain (*p* < 0.05): values labeled with **“a”** (good adherence) differ from those labeled **“b”** (poor adherence). Higher scores indicate a more supportive gluten-free food environment.

Participants with good adherence perceived significantly more supportive GFF environments than those with poor adherence (Figure 2). Differences were most pronounced in the home environment (weighted scores; median 4.79 vs. 1.29; *p* < 0.01), with a moderate effect size (Cohen’s *d* = 0.47; *r* = 0.26). The global NEMS-P-GF score also differed significantly between adherence groups (median 1.72 vs. −7.25; *p* < 0.01), showing a moderate effect (*d* = 0.55; *r* = 0.32). In contrast, the supply environment was consistently perceived as restrictive for both groups, showing a statistically significant difference (median −3.68 vs. −7.78; *p* = 0.018), with a small effect size (*d* = 0.29; *r* = 0.19). Comparable results were observed when non-weighted scores were used, with similar effect sizes and rank-biserial correlations across global, home, and supply domains (Supplementary Tables S3 and S4).

## 4. Discussion

This study aimed to adapt the Gluten-Free Perceived Nutrition Environment Measures Survey (NEMS-P-GF) for adults with CeD in Chile and provide a preliminary psychometric evaluation focused on content validity evidence, structural validity, and internal consistency. The adapted instrument demonstrated acceptable to excellent psychometric properties across retained domains, including adequate structural validity and internal consistency, supporting its use for assessing perceived GFF environments in this population.

As a secondary, exploratory objective, we examined associations between perceived GFF environments and adherence to a GFD to illustrate the instrument’s construct validity and applied relevance. Individuals with good adherence consistently perceived more supportive home and overall food environments than those with poor adherence, underscoring the role of environmental factors in sustaining dietary management for chronic conditions. In contrast, the supply environment was broadly perceived as less supportive, characterized by limited accessibility and higher prices of GFF. Together, these findings indicate that the NEMS-P-GF captures meaningful variation in environmental supportiveness relevant to the nutritional management of celiac disease.

Our results align with national and international evidence from observational and perception-based studies showing that GFF tends to be less available, less diverse, and more expensive than gluten-containing counterparts [41,42,43]. In Chile, similar challenges have been documented, with GFF being less accessible across retail outlets and costing up to three times more than conventional options [44]. By incorporating these structural challenges into the assessment of perceived environments, the NEMS-P-GF provides a more nuanced understanding of how affordability and accessibility shape adherence among adults with CeD and supports the instrument’s construct validity by reflecting well-documented contextual constraints.

Consistent with research using the NEMS-P framework, the home environment is perceived as the most supportive domain. Prior studies have emphasized the home as a protective setting for shaping dietary intake, including among individuals with CeD [31]. Evidence from the Chilean NEMS-P-Ch similarly indicates that domestic environments tend to be perceived as the least obesogenic, whereas supply and street environments are experienced as more obesogenic [40]. Extending this framework to a medically prescribed diet, our findings suggest that the home functions as a controlled space where risks of inadvertent gluten exposure and cross-contamination are minimized, a pattern also observed in qualitative studies, where individuals describe the home as a “sanctuary” from inadvertent gluten exposure [45]. However, this protective role is ultimately constrained by conditions in the broader supply environment. When access to GFF is limited or costly, households cannot maintain adequate availability, reflecting a dynamic where structural barriers directly affect domestic environments [32,46].

In contrast, the supply environment was consistently rated as unsupportive by both adherence groups. Limited variety, restricted availability of gluten-free processed products, and elevated prices resemble conditions described in the literature on “food deserts” or “food swamps,” where access to healthy or necessary foods is structurally constrained [47,48]. As a result, the limited availability and affordability of medically necessary foods can exacerbate dietary inequities, particularly among individuals with lower socioeconomic status, for whom the financial burden of GF products is particularly consequential. Collectively, these findings reinforce existing evidence indicating that environmental inequities, rather than individual motivation or preference, represent major constraints on optimal adherence to a GFD.

An additional consideration relates to the nutritional quality of GF products. Although expanding access to certified gluten-free foods is crucial for adherence, many commercially available options remain ultra-processed and nutritionally poor [49]. Increasing availability without addressing nutritional quality risks reproduces dynamics similar to those observed in obesogenic environments [50]. This underscores the need for public health policies that ensure both safety and nutritional adequacy, promoting healthier, minimally processed GF alternatives.

Given these findings, structural interventions are essential. Approaches centered exclusively on individual education or motivation are unlikely to succeed when the broader food environment remains unsupportive [51,52]. Public policies should therefore aim to guarantee access to safe and affordable gluten-free foods as part of the right to adequate nutrition [53]. Potential actions include subsidies or tax exemptions for GF products, strengthened labeling and cross-contamination controls, and expansion of specialized health services for adults with CeD [54]. Internationally, few countries offer financial support for GFD, most of them in Europe [54]. The United Kingdom’s GF prescription program, for example, has been associated with improved quality of life and reduced inequalities among beneficiaries [55].

In Chile, adults with CeD currently receive no financial support. In contrast, since 2022 the national food assistance program has expanded coverage for schoolchildren with CeD, providing daily GF meals in state-funded schools and a monthly food basket for home preparation. However, no equivalent program exists for the adult CeD population [56]. Until recently, certification of GF products was primarily managed by civil society organizations; under the new regulation on labeling, marketing, and sales, manufacturers are now responsible for demonstrating compliance with the national gluten threshold of 5 mg/kg [57]. Despite these advances, significant gaps persist in ensuring equitable access to safe and affordable GFF. The absence of comprehensive policies for adults with CeD represents a missed opportunity to promote adherence and prevent long-term complications. Broader calls within public health nutrition emphasize the need for systemic, multisectoral interventions involving health, agriculture, food industry, and local governance sectors to reduce structural barriers and promote equitable access to safe, healthy foods [58,59].

### Strengths and Limitations

The NEMS-P-GF constitutes the first adapted instrument with preliminary psychometric evidence to evaluate perceptions of GFF environments among adults with CeD. Its psychometric performance was acceptable to excellent across most domains, closely mirroring the internal consistency observed in the NEMS-P-Ch [33]. By adapting factor-weighted scoring to the GF context, this study operationalizes the construct of gluten-free food environment supportiveness for individuals with CeD, bridging clinical and public health nutrition frameworks. The integration of home- and supply-level perceptions provides a comprehensive understanding of the structural determinants of dietary management in chronic disease. Importantly, this represents the first systematic effort in Chile to measure GFF environments among adults with CeD, establishing a foundation for future research and policy action focused on environmental determinants of dietary management in chronic disease.

Several limitations should be acknowledged. Recruitment through patient advocacy organization’s social media channels and the voluntary, online nature of the survey may have introduced self-selection and coverage bias, as individuals who were more motivated, digitally connected, or health-conscious may have been more likely to participate. However, evidence suggests that the salience of the survey topic in recruitment materials does not necessarily influence respondents’ attitudes or behaviors in online surveys [60]. The demographic profile of the sample—characterized by a higher proportion of women and individuals with higher socioeconomic and educational levels—may therefore limit representativeness; this pattern is common in self-administered online health surveys [61]. Alternative recruitment strategies were considered but were not feasible within the study’s scope and resources, and post-stratification weighting was not possible due to the absence of national registries or population estimates for adults with CeD in Chile. In addition, the factor structure, scoring weights, and adherence associations were derived and tested within the same sample, which may introduce sample-specific optimization. Zero or weak loadings may reflect under-specification of complex domains (e.g., commensality). Future refinement should expand item coverage (e.g., commensality across meals and contexts) and reassess the appropriate measurement model and scoring. Sensitivity analyses using non-weighted NEMS-P-GF scores yielded comparable effect sizes and rank-biserial correlations, suggesting that observed associations were not driven by factor-derived weighting. Although exploratory adjusted analyses were performed, residual confounding cannot be excluded, and associations between perceived food environments and adherence should therefore be interpreted as descriptive rather than causal. Future studies should confirm these findings using independent samples and longitudinal designs.

Adherence to a GFD was assessed through self-reported questionnaires rather than objective clinical measures such as serological testing, histological evaluation, or detection of gluten immunogenic peptides, which are recommended best practices for monitoring established CeD [62]. Although self-reported instruments are widely used in population-based research, they may be subject to recall and social-desirability bias and may not fully capture inadvertent gluten exposure [63]. Nonetheless, online recruitment allowed us to reach a larger and more heterogeneous group in terms of disease trajectory and treatment experience than would have been possible through traditional clinical recruitment.

Additional limitations include the cross-sectional design, which precludes causal inference regarding the relationship between perceived food environments and adherence to a GFD. Because both the outcome (CDAT) and perceived environment predictors were self-reported within the same survey administration, common-method variance and differential reporting by adherence status may have biased the observed associations; moreover, the cross-sectional design provides no temporal ordering, so reverse causation cannot be ruled out. As data were collected at a single time point, we could not estimate test–retest reliability (which requires repeat administration under stable conditions) or measurement error; these properties should be evaluated in follow-up studies using a predefined retest interval (e.g., 7–14 days) and a consistent administration mode. Anthropometric and dietary information was self-reported, which may introduce reporting bias and measurement error. We did not collect information on clinical complications (e.g., anemia, elevated liver enzymes), limiting our ability to describe disease severity or examine whether complications are associated with adherence patterns. Finally, the current version of the NEMS-P-GF does not capture restaurant, street, or institutional food environments, which are relevant domains for future instrument extension and validation.

Future research should prioritize recruiting larger and more socioeconomically diverse samples to enhance representativeness. Incorporating objective dietary assessment by trained dietitians, alongside serological testing and gluten immunogenic peptide detection, could strengthen adherence evaluation. Furthermore, integrating psychosocial factors such as stress, well-being, social support, and stigma may provide a more nuanced understanding of how environmental and individual determinants interact. Longitudinal or mixed-methods designs will be essential to determine whether improvements in gluten-free food environments translate into measurable gains in dietary adherence and health outcomes.

## 5. Conclusions

This study provides empirical evidence that perceived food environment plays a key role in maintaining adherence to a GFD among Chilean adults with CeD. The NEMS-P-GF showed preliminary evidence of measurement adequacy—content validity evidence, structural validity, and internal consistency—for assessing perceived GFF environments in this population. Using this instrument, we found that more supportive home environments, characterized by greater availability of GFF, were associated with better adherence, whereas supply environments remained restrictive, particularly due to limited access and higher prices of GFF. These findings underscore the need for structural interventions that ensure the availability, accessibility, affordability, and safe access to GFF as a fundamental component of the right to adequate nutrition for people with CeD. Overall, this study contributes to public health strategies aimed at promoting more equitable and favorable food environments, particularly within the Chilean food supply, for individuals with CeD.

## Figures and Tables

**Table 1:** Dimensions of the adapted NEMS-P-GF.

Sub-Environment Measured	Variables	No. of Items
A. Home food environment	Availability of gluten-free and gluten-containing foods within the household; kitchen appliances used for food preparation; family commensality.	19
B. Food supply environment	Accessibility and affordability of gluten-free and gluten-containing products in frequently visited and neighborhood food stores; motivations for selecting food stores; importance assigned to various food attributes when purchasing food.	39

**Table 2:** Sociodemographic, anthropometric, and clinical characteristics of participants by adherence to a gluten-free diet (n = 233).

Variable	Good Adherence (CDAT < 13)	Poor Adherence (CDAT ≥ 13)	Total
Age (years), mean (SD)	40.3 (11.74) (n = 122)	36.8 (10.08) (n = 111)	38.6 (11.13) (n = 233)
Age group, n (%)			
18–35 years old	50 (41%)	55 (49.5%)	105 (45.1%)
36–59 years old	64 (52.5%)	54 (48.6%)	118 (50.6%)
61 years or older	8 (6.6%)	2 (1.8%)	10 (4.3%)
Gender, n (%)			
Female	107 (89.2%)	101 (91%)	208 (90%)
Male	12 (10%)	10 (9%)	22 (9.5%)
Sex, n (%)			
Women	110 (90.2%)	102 (91.9%)	212 (91%)
Men	12 (9.8%)	9 (8.1%)	21 (9%)
Participant’s educational level, n (%)			
Completed or less than high school	8 (6.6%)	12 (10.9%)	20 (8.6%)
Incomplete technical or university	36 (29.5%)	24 (21.8%)	60 (25.9%)
Completed university	45 (36.9%)	42 (38.2%)	87 (37.5%)
Postgraduate degree	33 (27%)	32 (29.1%)	65 (28%)
Household socioeconomic level, n (%)			
High	83 (71.6%)	69 (64.5%)	152 (68.2%)
Medium	23 (19.8%)	30 (28%)	53 (23.8%)
Low	10 (8.6%)	8 (7.5%)	18 (8.1%)
BMI (kg/m^2^), mean (SD)	24.3 (4.12) (n = 121)	25.5 (4.56) (n = 110)	24.8 (4.37) (n = 231)
Time since diagnosis, n (%)			
<3 years	47 (38.5%)	54 (48.6%)	101 (43.3%)
≥3 years	75 (61.5%)	57 (51.4%)	132 (56.7%)
Another household member with CeD, n (%)			
Yes	20 (16.4%)	12 (10.8%)	32 (13.7%)
No	97 (79.5%)	97 (87.4%)	194 (83.3%)
Don’t know	5 (4.1%)	2 (1.8%)	7 (3%)
Total, n (%)	122 (52.4%)	111 (47.6%)	233 (100%)

Note. CeD: Celiac disease; BMI: Body Mass Index; SD: Standard deviation; CDAT: Celiac Dietary Adherence Test. No significant differences were observed between adherence groups for sociodemographic, anthropometric, or clinical variables (all *p* > 0.05).

**Table 3:** Cronbach’s alpha and McDonald’s ω by NEMS-P-GF dimensions.

Construct (Question No.)	Description	No. of Items	Scale Range	Cronbach’s Alpha	McDonald’s ω
(A) Home food environment					
(A1) Gluten-free food availability at home					
Availability to GFF (18A–18J)	How often do you have … available in your home?	10	4	0.728	0.741
18A–18B	– Naturally gluten-free foods (FV on the fridge, FV on the counter)	2	4	0.622	0.636
18D,18F,18H,18J	– Gluten-free processed products (bread, pasta, sweets, biscuits, snacks, ice-cream, cakes, etc.)	4	4	0.703	0.706
18C,18E,18G,18I	– Gluten-containing processed products (bread, pasta, sweets, biscuits, snacks, ice-cream, cakes, etc.)	4	4	0.876	0.878
(B) Supply food environment					
(B1) Neighborhood accessibility to gluten-free food					
Ease of finding GFF (19A–19F)	Ease of finding certain foods in the district or neighborhood where you live	6	4	0.787	0.791
19A–19C	– Easy to find, good quality and selection: fresh produce and FV	3	4	0.902	0.906
19D–19F	– Easy to find, good quality and selection: GFF	3	4	0.901	0.903
(B2) Importance of choosing food stores					
Motivation selection place of purchase (20A–20G)	Motivation selection place of purchase	7	4	0.645	0.707
20A–20C	– Proximity to home, or on the way to passing sites, friends or family buy there	3	4	0.638	0.712
20D–20F	– Selection of foods, food quality, food prices	3	4	0.730	0.750
(B3) Food store accessibility to gluten-free food					
Accessibility buying GFF (21A–21K)	How easy or difficult is it to obtain … in the place where you buy most of your food?	11	4	0.796	0.790
21A–21C	– Naturally gluten-free foods (FV on the fridge, FV on the counter)	3	4	0.764	0.776
21E,21G,21I,21K,21M	– Gluten-free processed products (bread, pasta, sweets, biscuits, snacks, ice-cream, cakes, etc.)	4	4	0.904	0.905
21D,21F,21H,21J	– Gluten-containing processed products (bread, pasta, sweets, biscuits, snacks, ice-cream, cakes, etc.)	4	4	0.908	0.922
(B4) Food store gluten-free food prices					
Prices GFF (22A–22K)	How do you find the prices of … where you buy most of your food?	11	4	0.653	0.743
22A–22C	– Naturally gluten-free foods (FV on the fridge, FV on the counter)	3	4	0.768	0.780
22E,22G,22I,22K,22M	– Gluten-free processed products (bread, pasta, sweets, biscuits, snacks, ice-cream, cakes, etc.)	4	4	0.886	0.893
22D,22F,22H,22J	– Gluten-containing processed products (bread, pasta, sweets, biscuits, snacks, ice-cream, cakes, etc.)	4	4	0.914	0.915

## Data Availability

The data presented in this study are available within the article and the Supplementary Materials. The raw data supporting the conclusions of this article will be made available by the authors on request.

## Supplementary Materials

The following supporting information can be downloaded at: https://www.mdpi.com/article/10.3390/nu18060929/s1, Table S1. Factorial weights and weighted and non-weighted scores for the gluten-free food environments items; Table S2. Descriptive statistics of factors obtained for each food environment; Table S3. Home, supply, and global NEMS-P-GF according to GFD adherence categories (non-weighted scores); Table S4. Home, supply, and global NEMS-P-GF according to GFD adherence categories (weighted scores). Questionnaire—“Perceived Gluten-Free Food Environments and Adherence to a Gluten-Free Diet among Adults with Celiac Disease in Chile”.

## Abbreviations

The following abbreviations are used in this manuscript:

CeD	Celiac disease
GF	Gluten-free
GFD	Gluten-free diet
GFF	Gluten-free food
NEMS-P	Perceived Nutrition Environment Measures Survey
NEMS-P-Ch	Chilean Perceived Nutrition Environment Measures Survey
NEMS-P-GF	Gluten-free Perceived Nutrition Environment Measures Survey
