# Supplementary material for: Adaptation and Validation of the Gluten-Free Perceived Nutrition Environment Measures Survey (NEMS-P-GF) and Its Association with Gluten-Free Diet Adherence Among Adults with Celiac Disease in Chile
Source: Nutrients. 2026 Mar 16;18(6):929. doi: 10.3390/nu18060929 (PMC13028700; doi:10.3390/nu18060929)
Supplement: Supplementary file 1 [file nutrients-18-00929-s001.zip › 260301- Supplementary material_Questionnaire.pdf]

Questionnaire – “Perceived Gluten-Free Food Environments and Adherence to a Gluten-Free Diet among Adults with Celiac Disease in Chile” (English Translation)

Vega-Salas, M.J.; Parada, A.; Hermosilla-Llanca, D.; Rodríguez Osiac, L.; Rojas Egaña, D.; Berkowitz, L.; Rigotti, A.
*For the Spanish version, please request it from the authors directly.

General Background

To begin, I will ask you some general questions.

1. What is your sex?

a) Female

b) Male

c) Prefer not to answer

2. Which gender do you identify with?

a) Feminine

b) Masculine

c) Transmasculine

d) Transfeminine

e) Non-binary

f) Other. Please specify: __________

g) I don’t know

h) Prefer not to answer

3. What is your age?

____ years

4. What is the highest level of education you have completed (last year approved)?

Please select only one option.

a) No formal education

b) Completed primary education / incomplete or complete elementary or preparatory education

c) Completed secondary education (academic or technical–professional)

d) Completed technical institute (CFT) or professional institute (programs of 1 to 3 years) / up to non-commissioned officer in the Armed Forces or Police

e) Incomplete university education (programs of 4 or more years)

f) Completed university education (programs of 4 or more years) / commissioned officer in the Armed Forces or Police

g) Postgraduate education (postgraduate diploma, master’s degree, doctorate)

5. What is your height? (cm)*

____ cm

6. What is your weight? (kg)*

____ kg

*If you do not know or do not remember, please provide an approximate value.

7. Have you been diagnosed with celiac disease by a physician?

a) Yes

b) No → End survey

c) I am not sure → End survey

8. For the diagnosis of celiac disease, were endoscopic biopsy tests or blood tests detecting anti-endomysial or anti–tissue transglutaminase antibodies used?

a) Yes

b) No → End survey

c) I am not sure → End survey

9. How long ago were you diagnosed with celiac disease?

a) Less than 1 year ago

b) Between 1 year and less than 3 years ago

c) Between 3 years and less than 5 years ago

d) More than 5 years ago

Celiac Dietary Adherence Test (CDAT)

10. Have you been bothered by low energy level during the past 4 weeks?

a) None of the time

b) A little of the time

c) Some of the time

d) Most of the time

e) All the time

11. Have you been bothered by headaches during the past 4 weeks?

a) None of the time

b) A little of the time

c) Some of the time

d) Most of the time

e) All the time

Using the following scale, how much do you agree or disagree with the following statements?

	Strongly agree	Partially agree	Neither agree nor disagree	Partially disagree	Strongly disagree
**12. I am able to follow a GFD when dining outside my home**					
**13. Before I do something I carefully consider the consequences**					
**14. I do not consider myself a failure**					

15. How important to your health are accidental gluten exposures?
a) Very important
b) Somewhat important
c) Neutral/unsure
d) A little important
e) Not important at all

16. Over the past 4 weeks, how many times have you eaten foods containing gluten on purpose?
a) 0 (never)
b) 1–2
c) 3–5
d) 6–10
e) More than 10

END CDAT

---

17. Has any member of your household been diagnosed with celiac disease?
a) Yes
b) No
c) I don't know

Gluten-Free Perceived Nutrition Environment Measures Survey (NEMS-P-GF)

How often do you have the following foods available in your home?

	Never or rarely	Sometimes	Often	Almost always
**18A. Fruits and/or vegetables in the refrigerator**				
**18B. Fruits and/or vegetables kept out in the open (in a bowl or on the counter)**				
**18C. Bread WITH GLUTEN**				
**18D. Bread WITHOUT GLUTEN**				
**18E. Pasta WITH GLUTEN**				
**18F. Pasta WITHOUT GLUTEN**				
**18G. Packaged sweets/candies, cookies, chips, or salty snacks WITH GLUTEN**				
**18H. Packaged sweets/candies, cookies, chips, or salty snacks WITHOUT GLUTEN**				
**18I. Ice cream, cakes, pastries, or fresh baked goods (e.g., cake, brownies, muffins, etc.) WITH GLUTEN**				
**18J. Ice cream, cakes, pastries, or fresh baked goods (e.g., cake, brownies, muffins, etc.) WITHOUT GLUTEN**				

Please indicate your level of agreement or disagreement with each statement.

	Strongly agree	Partially agree	Partially disagree	Strongly disagree
**19A. It is easy to buy fresh fruits and vegetables in my neighborhood**				
**19B. The fresh products available in my neighborhood are of good quality**				
**19C. There is an excellent selection of fresh fruits and vegetables in my neighborhood**				
**19D. It is easy to buy gluten-free products in my neighborhood**				
**19E. The gluten-free products available in my neighborhood are of good quality**				

**19F. There is an excellent selection of gluten-free products in my neighborhood**				

How important are the following factors when choosing where you do most of your food shopping?

	Not at all important	A little important	Somewhat important	Very important
**20A. Proximity to my home**				
**20B. Proximity to my usual route or to other places I frequently visit**				
**20C. Friends or family members who shop at the same place**				
**20D. Selection of foods**				
**20E. Quality of foods**				
**20F. Prices of foods**				
**20G. Access to public transportation**				

In the place where you buy most of your food, how easy or difficult is it to access the following types of foods?

	Very easy	Somewhat easy	Somewhat difficult	Very difficult
**21A. Fresh fruits and vegetables**				
**21B. Frozen or packaged fruits and vegetables (cans or boxes)**				
**21C. Lean meats (low fat)**				
**21D. Bread WITH GLUTEN**				
**21E. Bread WITHOUT GLUTEN**				
**21F. Pasta WITH GLUTEN**				
**21G. Pasta WITHOUT GLUTEN**				
**21H. Packaged sweets/candies, cookies, chips, or salty snacks WITH GLUTEN**				
**21I. Packaged sweets/candies, cookies, chips, or salty snacks WITHOUT GLUTEN**				
**21J. Ice cream, cakes, pastries, or fresh baked goods (e.g., cake, brownies, muffins, etc.) WITH GLUTEN**				
**21K. Ice cream, cakes, pastries, or fresh baked goods (e.g., cake, brownies, muffins, etc.) WITHOUT GLUTEN**				

Regarding the place where you buy most of your food, how would you rate the price of the following items?

	Very inexpensive	Not expensive	Somewhat expensive	Very expensive
**22A. Fresh fruits and vegetables**				
**22B. Frozen or packaged fruits and vegetables (cans or boxes)**				
**22C. Lean meats (low fat)**				
**22D. Bread WITH GLUTEN**				
**22E. Bread WITHOUT GLUTEN**				
**22F. Pasta WITH GLUTEN**				
**22G. Pasta WITHOUT GLUTEN**				
**22H. Packaged sweets/candies, cookies, chips, or salty snacks WITH GLUTEN**				
**22I. Packaged sweets/candies, cookies, chips, or salty snacks WITHOUT GLUTEN**				
**22J. Ice cream, cakes, pastries, or fresh baked goods (e.g., cake, brownies, muffins, etc.) WITH GLUTEN**				
**22K. Ice cream, cakes, pastries, or fresh baked goods (e.g., cake, brownies, muffins, etc.) WITHOUT GLUTEN**				

When you buy food, how important is each of the following to you?

	Not at all important	Somewhat important	Very important
**23A. Taste**			
**23B. Nutritional quality**			
**23C. Price**			
**23D. Convenience**			

	Never	Occasionally	Sometimes	Almost always or always
**24. How often do you prepare meals different from the rest of your family because of your celiac disease?**				
**25. How often does your family eat meals together?**				
**26. How often does your family eat meals in front of the television, computer, phone, or another electronic device that is turned on?**				

Do you have the following appliances for cooking or storing food in your home?

	**Yes**	**No**
**27A. Refrigerator**		
**27B. Freezer**		

**27C. Microwave**		
**27D. Stove**		
**27E. Oven**		
**27F. Other cooking appliances**		

Sociodemographic Characteristics (Final)

28. What is your nationality?

a) Chilean
b) Venezuelan
c) Peruvian
d) Colombian
e) Bolivian
f) Argentine
g) Other. Please specify: _____________

29. Do you consider yourself to belong to an Indigenous or Native people?

a) Yes. Which one? _____________
b) No

30. What is your current marital status?

a) Married
b) Cohabiting or in a partnership without a civil union agreement
c) Civil partnership (with a civil union agreement)
d) Annulled
e) Separated
f) Widowed
g) Single

Socioeconomic Characterization Survey (AIM)

31. What is the highest level of education attained (last year completed) by the main household breadwinner?

Please select only one option.
h) No formal education
i) Incomplete primary or elementary education
j) Completed primary or elementary education
k) Incomplete secondary education (academic or technical–professional)
l) Completed secondary education (academic or technical–professional)
m) Incomplete technical institute (CFT) or professional institute (programs of 1 to 3 years)
n) Completed technical institute (CFT) or professional institute (programs of 1 to 3 years) / up to non-commissioned officer in the Armed Forces or Police
o) Incomplete university education (programs of 4 or more years)

p) Completed university education (programs of 4 or more years) / commissioned officer in the Armed Forces or Police
q) Postgraduate education (postgraduate diploma or master's degree, master's, doctorate)
r) I don't know

32. Which of the following occupations corresponds to the job of the main household breadwinner? (If the main household breadwinner is unemployed or retired, ask about their last paid occupation. If the breadwinner has more than one job, record the one with the highest income.)

Please select only one option.

a) Senior executives (general manager or area/sector manager) of private or public companies; directors or owners of large companies; senior officials of the executive branch, legislative bodies, or public administration (includes commissioned officers of the Armed Forces or Police)
b) Professionals, scientists, and intellectuals
c) Technicians and associate professionals (includes up to non-commissioned officers of the Armed Forces/Police)
d) Clerical support workers in public and private offices
e) Service workers and salespersons in commerce and markets
f) Skilled agricultural, forestry, and fishery workers
g) Craft and related trades workers, machine operators, and artisans of mechanical and other trades
h) Plant and machine operators, assemblers, drivers, and vehicle operators
i) Elementary occupations in sales and services; agricultural, forestry, and construction laborers, etc.
j) Other non-classified groups (includes rentiers, persons with disabilities, etc.)
k) I don't know

33. Including yourself, how many people currently live in your household? (Do not include domestic service staff, even if they live in.)

Please think about your household's total income in an average month, considering the contribution of all members and other additional income such as property rents, pensions, or retirement benefits. Into which of the following income brackets does your total monthly household income fall? **Apply the category according to your answer in Question 3.**

a) 1
b) 2
c) 3
d) 4
e) 5
f) 6
g) 7
h) Don't know

1 Member	Total household income range
$0 - $124K CLP	1
$125K - $217K CLP	2
$218K - $379K CLP	3
$380K - $661K CLP	4
$662K - $1.1M CLP	5
$1.2M- $2.0M CLP	6
$2.1M + CLP	7

2 Members	Total household income range
$0 - $201K CLP	1
$202K - $353K CLP	2
$354K - $616K CLP	3
$616K - $1.0M CLP	4
$1.1M- $1.8M CLP	5
$1.9M- $3.2M CLP	6
$3.3M + CLP	7

3 Members	Total household income range
$0 - $268K CLP	1
$269K - $469K CLP	2
$470K - $818K CLP	3
$819K - $1.4M CLP	4
$1.5M- $2.4M CLP	5
$2.5M- $4.3M CLP	6
$4.4M + CLP	7

4 Members	Total household income range
$0 - $328K CLP	1
$329K - $574K CLP	2
$575K - $1.0M CLP	3
$1.1M - $1.7M CLP	4
$1.8M- $3.0M CLP	5
$3.1M- $5.3M CLP	6
$5.4M + CLP	7

5 Members	Total household income range
$0 - $384K CLP	1
$385K - $671K CLP	2
$672K - $1.1M CLP	3
$1.2M - $2.0M CLP	4
$2.1M- $3.5M CLP	5
$3.6M- $6.2M CLP	6
$6.3M + CLP	7

6 Members	Total household income range
$0 - $436K CLP	1
$437K - $762K CLP	2
$763K - $1.3M CLP	3
$1.4M - $2.3M CLP	4
$2.4M- $4.0M CLP	5
$4.1M- $7.0M CLP	6
$7.1M + CLP	7

7 Members	Total household income range
$0 - $486K CLP	1
$487K - $849K CLP	2
$850K - $1.4M CLP	3
$1.5M - $2.5M CLP	4
$2.6M- $4.5M CLP	5
$4.6M- $7.8M CLP	6
$7.9M + CLP	7
