# Supplementary material for: Adaptation and Validation of the Gluten-Free Perceived Nutrition Environment Measures Survey (NEMS-P-GF) and Its Association with Gluten-Free Diet Adherence Among Adults with Celiac Disease in Chile
Source: Nutrients. 2026 Mar 16;18(6):929. doi: 10.3390/nu18060929 (PMC13028700; doi:10.3390/nu18060929)
Supplement: Supplementary file 1 [file nutrients-18-00929-s001.zip › 260301- Supplementary material_Tables.pdf]

Supplementary material – Tables

Table S1. Factorial weights and weighted and non-weighted scores for the gluten-free food environments items

Factor	Question	Factorial weight (EFA)	Non-weighted score		Weighted score	
			Min	Max	Min	Max
**(A) Home food environment**						
**(A1) Gluten-free food availability at home (KMO=0.712; Barlett χ^2=825; p=<0.001)**						
F1. Availability of naturally GFF at home	18A. Fruits and vegetables on the fridge	0.595	-2	2	-1.190	1.190
	18B. Fruits and vegetables over the counter or table	0.671	-2	2	-1.342	1.342
F2. Availability of GFF-processed products at home	18D. GF Bread	0.558	-2	2	-1.116	1.116
	18F. GF Pasta	0.471	-2	2	-0.942	0.942
	18H. GF packed sweets, biscuits, crisps, salted or sweet snacks	0.747	-2	2	-1.494	1.494
	18J. GF ice cream, cakes, pastries, fresh baked goods	0.656	-2	2	-1.312	1.312
F3. Availability of gluten-containing processed products at home	18C. Bread	0.797	-2	2	-1.594	1.594
	18E. Pasta	0.816	-2	2	-1.632	1.632
	18G. Packed sweets, biscuits, crisps, salted or sweet snacks	0.855	-2	2	-1.71	1.71
	18I. Ice cream, cakes, pastries, fresh baked goods	0.76	-2	2	-1.520	1.520
**(A2) Kitchen appliances available at home (KMO=0.54; Barlett χ^2=46.7; p=<0.001)**						
F4. Kitchen appliances	27A. Refrigerator	0.253	-1	1	-0.253	0.253
	27B. Freezer	0.478	-1	1	-0.478	0.478
	27C. Microwave	0.445	-1	1	-0.445	0.445
	27D. Stove	0.16	-1	1	-0.16	0.16
	27E. Oven	0.229	-1	1	-0.229	0.229
	27F. Other cooking appliances	0.259	-1	1	-0.259	0.259
**(A3) Family commensality (KMO=0.5; Barlett χ^2=5.06; p=0.917)**						
Frequency commensality tensions	24. How often prepare different dishes because celiac disease	0	-2	2	0	0
Frequency healthy commensality	25. How often eat meals with family	0	-2	2	0	0
Frequency unhealthy commensality	26. How often does the family eat in front of screens (TV, computer, smartphone, etc.)	0	-2	2	0	0
**(B) Store Food Environment**						
**(B1) Neighborhood accessibility gluten-free food (KMO=0.745; Barlett χ^2=915; p=<0.001)**						

F5. Ease of finding fresh naturally GFF at neighborhood	19A. It is easy to buy fresh fruits and vegetables in my neighborhood	0.797	-2	2	-1.594	1.594
	19B. The fresh products available in my neighborhood are of good quality	0.936	-2	2	-1.872	1.872
	19C. There is an excellent selection of fresh fruits and vegetables in my neighborhood	0.873	-2	2	-1.746	1.746
F6. Ease of finding GFF products at neighborhood	19D. It is easy to buy gluten-free products in my neighborhood	0.892	-2	2	-1.784	1.784
	19E. The gluten-free products available in my neighborhood are of good quality	0.804	-2	2	-1.608	1.608
	19F. There is an excellent selection of gluten-free products in my neighborhood	0.907	-2	2	-1.814	1.814
**(B2) Importance for choosing food stores (KMO=0.65; Barlett χ^2=360; p=<0.001)**						
F7. Importance of social and spatial proximity for choosing food stores	20A. Proximity to my home	0.766	-2	2	-1.532	1.532
	20B. Proximity to my usual route or other places I frequently visit	0.88	-2	2	-1.76	1.76
	20C. Friends or family members who shop at the same place	0.332	-2	2	-0.664	0.664
F8. Importance of food selection, quality, and price for choosing food stores	20D. Food selection	0.8	-2	2	-1.6	1.6
	20E. Food quality	0.744	-2	2	-1.488	1.488
	20F. Food prices	0.564	-2	2	-1.128	1.128
	20G. Access to public transportation	0	-2	2	0.000	0.000
**(B3) Food store accesibility gluten-free food (KMO=0.814; Barlett χ^2=1704; p=<0.001)**						
F9. Store accessibility of naturally GFF	21A. Fresh fruits and vegetables	0.595	-2	2	-1.19	1.19
	21B. Frozen or packaged fruits and vegetables (cans or boxes)	0.724	-2	2	-1.448	1.448
	21C. Lean meats (low fat)	0.762	-2	2	-1.524	1.524
F10. Store accessibility of GFF-processed products	21E. GF Bread	0.833	-2	2	-1.666	1.666
	21G. GF Pasta	0.867	-2	2	-1.734	1.734
	21I. GF packed sweets, biscuits, crisps, salted or sweet snacks	0.845	-2	2	-1.69	1.69
	21K. GF ice cream, cakes, pastries, fresh baked goods	0.716	-2	2	-1.432	1.432
F11. Store accessibility of gluten-containing processed products	21D. Bread	0.852	-2	2	-1.704	1.704
	21F. Pasta	0.889	-2	2	-1.778	1.778
	21H. Packed sweets, biscuits, crisps, salted or sweet snacks	0.989	-2	2	-1.978	1.978
	21J. Ice cream, cakes, pastries, fresh baked goods	0.739	-2	2	-1.478	1.478

**(B4) Food store gluten-free food prices (KMO=0.799; Barlett χ^2=1506; p=<0.001)**						
F12. Prices of naturally GFF	22A. Fresh fruits and vegetables	0.77	-2	2	-1.54	1.54
	22B. Frozen or packaged fruits and vegetables (cans or boxes)	0.845	-2	2	-1.69	1.69
	22C. Lean meats (low fat)	0.507	-2	2	-1.014	1.014
F13. Prices of GFF-processed products	22E. GF Bread	0.811	-2	2	-1.622	1.622
	22G. GF Pasta	0.748	-2	2	-1.496	1.496
	22I. GF packed sweets, biscuits, crisps, salted or sweet snacks	0.933	-2	2	-1.866	1.866
	22K. GF ice cream, cakes, pastries, fresh baked goods	0.763	-2	2	-1.526	1.526
F14. Prices of gluten-containing processed products	22D. Bread	0.732	-2	2	-1.464	1.464
	22F. Pasta	0.876	-2	2	-1.752	1.752
	22H. Packed sweets, biscuits, crisps, salted or sweet snacks	0.892	-2	2	-1.784	1.784
	22J. Ice cream, cakes, pastries, fresh baked goods	0.876	-2	2	-1.746	1.746
**(B5) Food attributes for choosing food stores (KMO=0.524; Barlett χ^2=186; p=<0.001)**						
F15. Food Purchase Attributes	23A. The taste	0.239	-1	1	-0,239	0,239
	23B. The nutritional quality	0.159	-1	1	-0,159	0,159
	23C. The price	0.701	-1	1	-0.701	0.701
	23D. That it is convenient	1.002	-1	1	-1.002	1.002

GF: Gluten-free; GFF: Gluten-free foods; EFA: exploratory factorial analysis. All factors <0.3 are presented as 0.

The item “23D. That it is convenient” displayed a factor loading >1.0, indicative of a Heywood-type case; consequently, this item and its corresponding factor were excluded from the final factor structure and were not included in the calculation of domain or global NEMS-P-GF scores.

Table S2. Descriptive statistics of factors obtained for each food environment

Factor	N	Non-weighted score						Weighted score					
		Mean	SD	Median	P25-P75	Min	Max	Mean	SD	Median	P25-P75	Min	Max
**Global NEMS-GF**	206	-1.41	15.99	-1.5	-13-10	-46	33	-2.83	12.56	-2.88	-12.006-6.521	-38.2	24.7
**(A) Home food environment**	226	5.21	8.23	5.21	0-12	-15	20	3.24	6.02	2.83	-0.871-8.379	-10.6	13.9
**(A1) Gluten-free food availability at home**													
F1. Availability of naturally GFF at home	232	1.966	2.27	3	0-4	-4	4	1.224	1.45	1.861	-0.076-2.532	-2.530	2.530
F2. Availability of GFF-processed products at home	231	2.234	4.21	3	-1-5	-8	8	1.217	2.61	1.591	-1.019-3.184	-4.86	4.86
F3. Availability of gluten-containing processed products at home	229	1.017	5.78	1	-4-7	-8	8	0.803	4.68	0.74	-3.321-5.659	-6.46	6.46
**(B) Supply food environment**	211	-6.71	12.77	-7	-15-2	-37	26	-6.14	10.43	-6.65	-12.93-0.574	-30.2	19.9
**(B1) Neighborhood accessibility to gluten-free food**													
F5. Ease of finding fresh naturally GFF at neighborhood	232	3.392	3.4	5	2.75-6	-6	6	2.945	2.95	4.339	2.4508-5.212	-5.21	5.21
F6. Ease of finding GFF products at neighborhood	229	-1.044	4.3	-2	-5-3	-6	6	-0.937	3.73	-1.902	-4.402-2.603	-5.21	5.21
**(B2) Importance of choosing food stores**													

F7. Importance of social and spatial proximity for choosing food stores	232	1.379	3	2	0-3	-6	6	1.557	2.04	2.08	0.982-2.96	-3.96	3.96
F8. Importance of food selection, quality, and price for choosing food stores	228	4.873	2.05	6	5-6	-6	6	3.43	1.45	4.216	3.416-4.216	-4.22	4.22
**(B3) Food store accessibility to gluten-free food**													
F9. Store accessibility of naturally GFF	231	4.394	2.32	5	3-6	-6	6	3.028	1.63	3.567	2.081-4.162	-4.16	4.16
F10. Store accessibility of GFF-processed products	232	-0.203	5.26	-1	-4-4	-8	8	-0.102	4.31	-0.577	-3.261-3.658	-6.52	6.52
F11. Store accessibility of gluten-containing processed products	231	-7.502	2.15	-8	-8- -8	-8	8	-6.515	1.86	-6.938	-6.938--6.938	-6.94	6.94
**(B4) Food store gluten-free food prices**													
F12. Prices of naturally GFF	230	-2.339	2.53	-3	-4- -1	-6	6	-1.547	1.85	-2.122	-2.629 - - 0.582	-4.24	4.24
F13. Prices of GFF-processed products	229	-6.638	2.22	-8	-8- -5	-8	8	-5.441	1.82	-6.554	-6.554--4.11	-6.55	6.55
F14. Prices of gluten-containing processed products	229	-3.21	4.1	-4	-6-0	-8	8	-2.719	3.47	-3.373	-5.141--0.163	-6.75	6.75

GF: Gluten-free; GFF: Gluten-free foods

Table S3. Home, supply, and global NEMS-GF according to GFD adherence categories (non-weighted scores)

Factor	N	Good adherence (<13 CDAT)						Poor adherence (≥13 CDAT)						p	Cohen's d	r (Mann–Whitney)
		Mean	SD	Median	P25-P75	Min	Max	Mean	SD	Median	P25-P75	Min	Max			
**Global NEMS-GF**	206	2.73	15.7	5	-10-15	-44	33	-5.71	15.2	-7	-15-3	-46	31	0.001	0.55	0.32
**(A) Home food environment**	226	7.03	7.54	7	2-13	-15	20	3.26	8.52	3	-3 - 9	-14	20	0.001	0.47	0.26
**(B) Supply food environment**	211	-5	13.3	-5	-14-7	-37	18	-8.53	12	-9	-16- -2.25	-36	26	0.02	0.28	0.19

GF: Gluten-free; GFF: Gluten-free foods; Mann-Whitney U test; weighted scores presented. Participants with good adherence perceived significantly more supportive global, home, and supply GFF environments than those with poor adherence.

Table S4. Home, supply, and global NEMS-GF according to GFD adherence categories (weighted scores)

Factor	N	Good adherence (<13 CDAT)						Poor adherence (≥13 CDAT)						p	Cohen's d	r (Mann–Whitney)
		Mean	SD	Median	P25-P75	Min	Max	Mean	SD	Median	P25-P75	Min	Max			
**Global NEMS-GF**	206	0.424	12.3	1.72	-9.12-10.6	-35	24.7	-6.22	11.9	-7.25	-14.4 - 0.186	-38.2	22.9	0.001	0.47	0.26
**(A) Home food environment**	226	4.58	5.56	4.79	-0.041-9.35	-10.6	13.9	1.8	6.18	1.29	-2.91-6.46	-9.26	13.9	0.018	0.29	0.19
**(B) Supply food environment**	211	-4.7	10.9	-3.68	-12.2-4.44	-30.2	15.2	-7.69	9.76	-7.78	-13.5--2.92	-29.8	19.9	0.001	0.55	0.32

GF: Gluten-free; GFF: Gluten-free foods; Mann-Whitney U test; weighted scores presented. Participants with good adherence perceived significantly more supportive global, home, and supply GFF environments than those with poor adherence
